# Topological Friction and Relaxation Dynamics of Spatially Confined Catenated Polymers

**DOI:** 10.1021/acsmacrolett.1c00594

**Published:** 2021-12-13

**Authors:** Giulia Amici, Michele Caraglio, Enzo Orlandini, Cristian Micheletti

**Affiliations:** † Scuola Internazionale Superiore di Studi Avanzati - SISSA, via Bonomea 265, 34136 Trieste, Italy; ‡ Institut für Theoretische Physik, Universität Innsbruck, Technikerstraße 21A, A-6020 Innsbruck, Austria; ¶ Department of Physics and Astronomy, University of Padova, Via Marzolo 8, I-35100 Padova, Italy

## Abstract

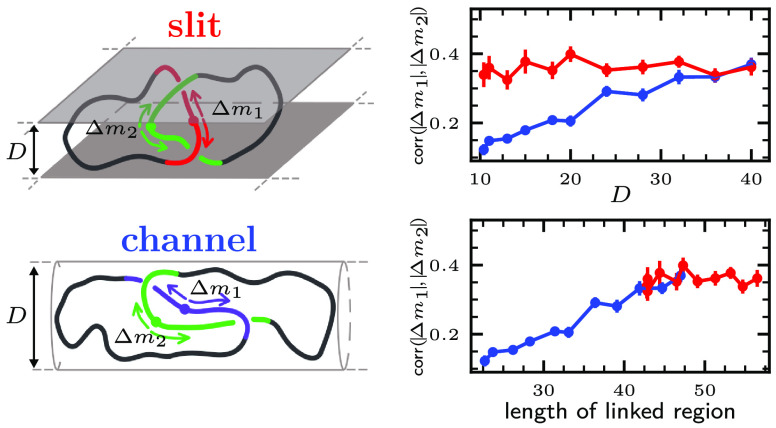

We study catenated ring polymers confined inside channels and slits with Langevin dynamics simulations and address how the contour position and size of the interlocked or physically linked region evolve with time. We show that the catenation constraints generate a drag, or topological friction, that couples the contour motion of the interlocked regions. Notably, the coupling strength decreases as the interlocking is made tighter, but also shorter, by confinement. Though the coupling strength differs for channel and slit confinement, the data outline a single universal curve when plotted against the size of the linked region. Finally, we study how the relaxation kinetics changes after one of the rings is cut open and conclude that considering interlocked circular polymers is key for isolating the manifestations of topological friction. The results ought to be relevant for linked biomolecules in experimental or biological confining conditions.

Mutual entanglement, or linking, between fluctuating filaments is ubiquitous in biological systems^1−4^ and is increasingly present in designed soft-matter systems, such as supramolecular constructs,^5−8^ olympic gels,^8−11^ and interlocked ssDNA rings.^12^

The convergent theoretical and experimental focus on interlocked molecular systems has given us much insight about the unusual physical properties of these systems, e.g., how they typically establish low-dimensional extended structures, how they elongate and flatten when sheared,^13−15^ and how they are affected by adsorbing surfaces^6^ or varying quality of the solvent.^16^ Multichain entanglement is also found in genomic DNA subject to anisotropic spatial confinement *in vivo*, such as intertwined sister nucleoids inside newly divided bacteria^17^ and intermingled chromosomes inside eukaryotic nuclei.^4^

Despite these relevant and common examples, linked ring polymers have been limitedly studied compared to polymeric systems with nonpermanent forms of entanglements, such as solutions or melts of linear polymers where the interplay of entanglement density and chain length has been extensively addressed.^18−21^

Little is known about the response of linked polymers to confinement, too. While the linking probability of confined rings has long been studied,^22,23^ there are no results yet for how metric properties of interlocked rings vary from one- to two-dimensional confinement. Also, we mostly ignore how the rings’ internal dynamics is differently affected by confinement and catenation constraints. This is partly due to the challenges posed by pinpointing the interlocked or physically linked regions or mutually entangled open or closed chains, for which algorithmic strategies combining geometrical and topological concepts have only recently become available.^24,25^

Here, we address these unexplored questions for the prototypic case of two Hopf-linked rings, which are evolved with a Langevin dynamics in one- and two-dimensional confinement. By using the methods of refs (24 and 25), we directly track interlocked regions and analyze how their contour lengths, , and contour migration kinetics vary with confinement. We establish that the Hopf link topological constraint introduces an effective coupling or topological friction, in the migration of the interlocked segments, expose the key role played by  across all types and degrees of considered confinement, and contrast the slow migration kinetics with the fast unthreading of one of the rings after cutting it open.

Our reference system consists of two catenated semiflexible rings, each of *N* = 360 beads, placed inside channels or slits of width *D*, see Figure 1a,b. The beads have nominal diameter σ, and their excluded volume interactions are accounted for by a truncated and shifted Lennard-Jones (LJ) potential. The same LJ term is used for the steric repulsion of the beads and the structureless walls of the channels and slits, which extend for a length of 400σ in the longitudinal direction(s), where periodic boundary conditions apply. Chain connectivity is provided by a FENE potential, and the chain bending rigidity is set to yield a nominal persistence length of *l*_*p*_ = 5σ. The Langevin dynamics of the system was integrated numerically with the LAMMPS simulation package^26^ with default values for the mass and friction coefficients^20^ and with an integration time step equal to 0.005τ_LJ_, where τ_LJ_ is the characteristic simulation time. At each value of *D*, we collected ten trajectories, each covering a time span of 10^7^τ_LJ_, which is much larger than the slowest relaxation times of the system, see the Supporting Information (SI).

**Figure 1:**
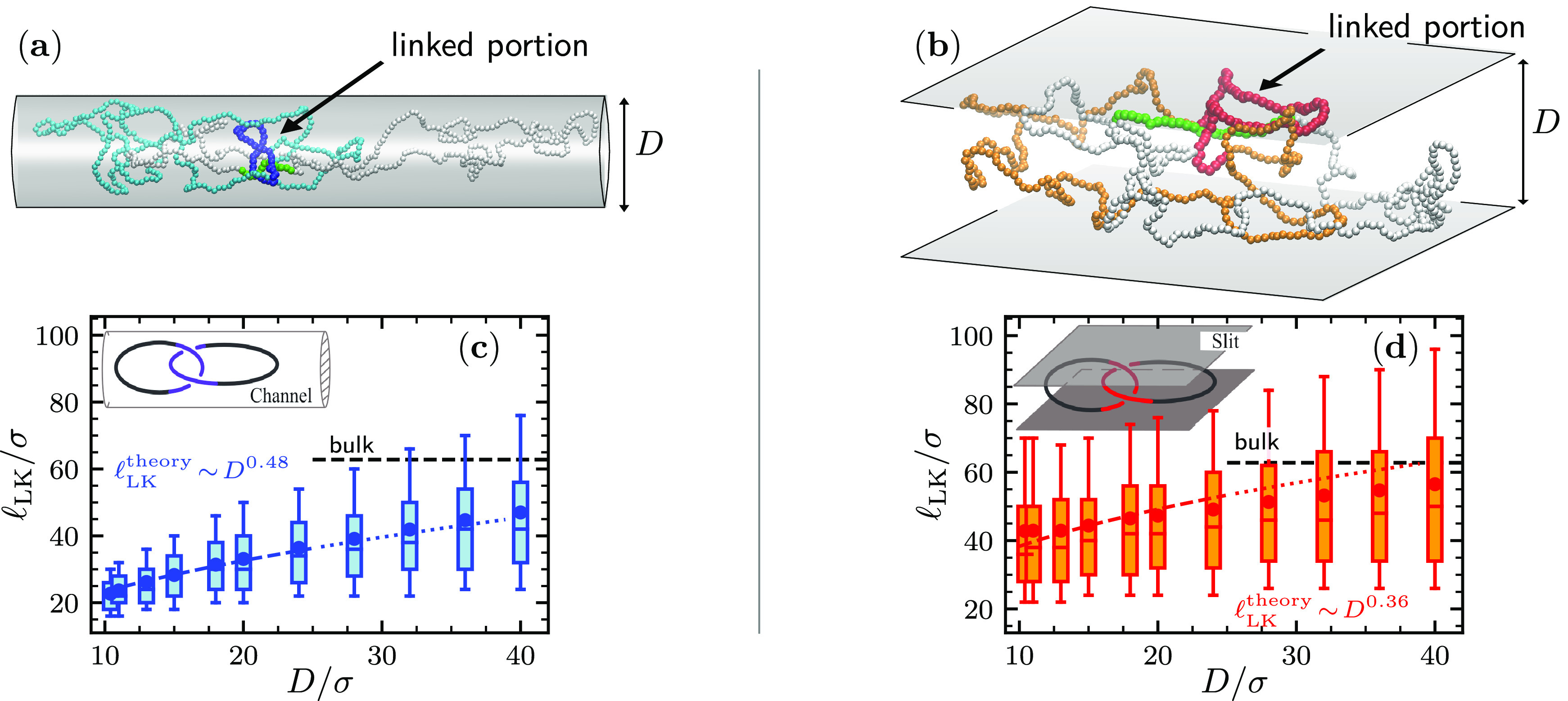
Typical configuration of semiflexible Hopf-linked rings inside a channel (a) and a slit (b) of width *D* = 20σ. Each ring comprises *N* = 360 beads of diameter σ and has a persistence length *l*_*p*_ = 5σ. The linked portion, highlighted with different saturated colors, consists of the shortest pairs of segments (one per ring) that yield a Hopf link topology upon closure, see the SI. (c,d) Box-whisker plots (point, average; center line, median; box limits, upper and lower quartiles; whiskers, 10th and 90th percentiles) of the linked portion length, , for different channel and slit widths. The curves are the best fits based on the expected scaling in the range of the anisometric blob regime for linear chains, 10 < *D*/σ < 25, marked by the dashed portion of the fitting curves. The horizontal dashed lines are for the unconstrained (bulk) case. More general scaling fits and with different ranges of the bare and effective confinement widths are presented in Figure S2.

The interlocked or linked region was identified using the strategy of ref (24), which uses a top-down search to single out the shortest (linear) portions of the two rings that yield the same Hopf topology of the whole system, once suitably closed. The closure is obtained by prolonging the termini of one portion away from the center of mass of the other and then bridging “at infinity”, see the SI.

We first discuss the effect of confinement dimensionality and degree on the static properties of the linked region and specifically on its size, , which is given by the summed contour lengths of the interlocked portions of the two rings. The results are given in Figure 1c,d. For both slits and channels, the average linked portion length, , decreases from the bulk value of 62 ± 2σ as *D* is reduced.

The considered range of confinement includes the anisometric blob regime of linear chains, which is expected in channels and slits of widths 2*l*_*p*_ ≲ *D* ≲ *l*_*p*_^2^/σ, where the blob length expectedly scales as l_*_ ∝ *D*^4/3^ for channels^27^ and as l_*_ ∝ *D* for slits.^28,29^ The implied scaling for the longitudinal span is *D*^–2/3^ for channels and *D*^–1/4^ for slits, which both apply for our Hopf links, too, see Figure S1.

Based on the ansatz that the entangled portion is sequestered inside two interlocked anisometric blobs,^30^ one can speculate that , where α = 0.36 ± 0.05 is the bulk exponent for unconstrained Hopf links.^24^ The argument yields  for slits and  for channels. As shown by the fit in Figure 1c, the latter relationship is in accord with the channel data, confirming previous results.^30^ On the other hand, data for slits deviate from the expected trend (see Figure 1d) and are best described by the power-law fit , see Figure S2b.

As a matter of fact, the average  levels off for slit widths about equal to the Kuhn length, 10σ, and only modest changes are observed in the corresponding  probability distributions compared to the channel case, see Figure S1(e,f). Thus, in slit confinement, the progressive longitudinal elongation of the rings is accompanied by a weaker tightening of the linked region compared to channels. We surmise that this could be related to entropic segregation forces^31−34^ being weaker in slits than in channels.

The sensitive dependence of the linked portion static properties on confinement extends to kinetics too. To address this, we considered how the two interlocked regions migrate along the rings’ contours over time spans of at least 10^6^τ_LJ_. The migration process appears to be diffusive, see Figures S3 and S4, and we studied it by tracking the chain indexes, *m*_1_ and *m*_2_, of the central monomers of the interlocked regions and computed their absolute increments, |*Δm*_1_| and |*Δm*_2_|, for a fixed time lag *Δt* = 100τ_LJ_.

Typical time evolutions of the |*Δm*|’s are presented in Figure 2a,b and establish two results. First, the |*Δm*|’s magnitude responds very differently to increasing confinement in channels and slits. Specifically, the average contour displacement reduces by a factor of 3 when the channel width is reduced from *D* = 40σ to *D* = 10σ, while for slits, this decrease is milder, see Figure 2c. Thus, at equal *D*, channels both shorten the physically linked regions more effectively than slits and also introduce a larger hindrance to their contour sliding motion.

**Figure 2:**
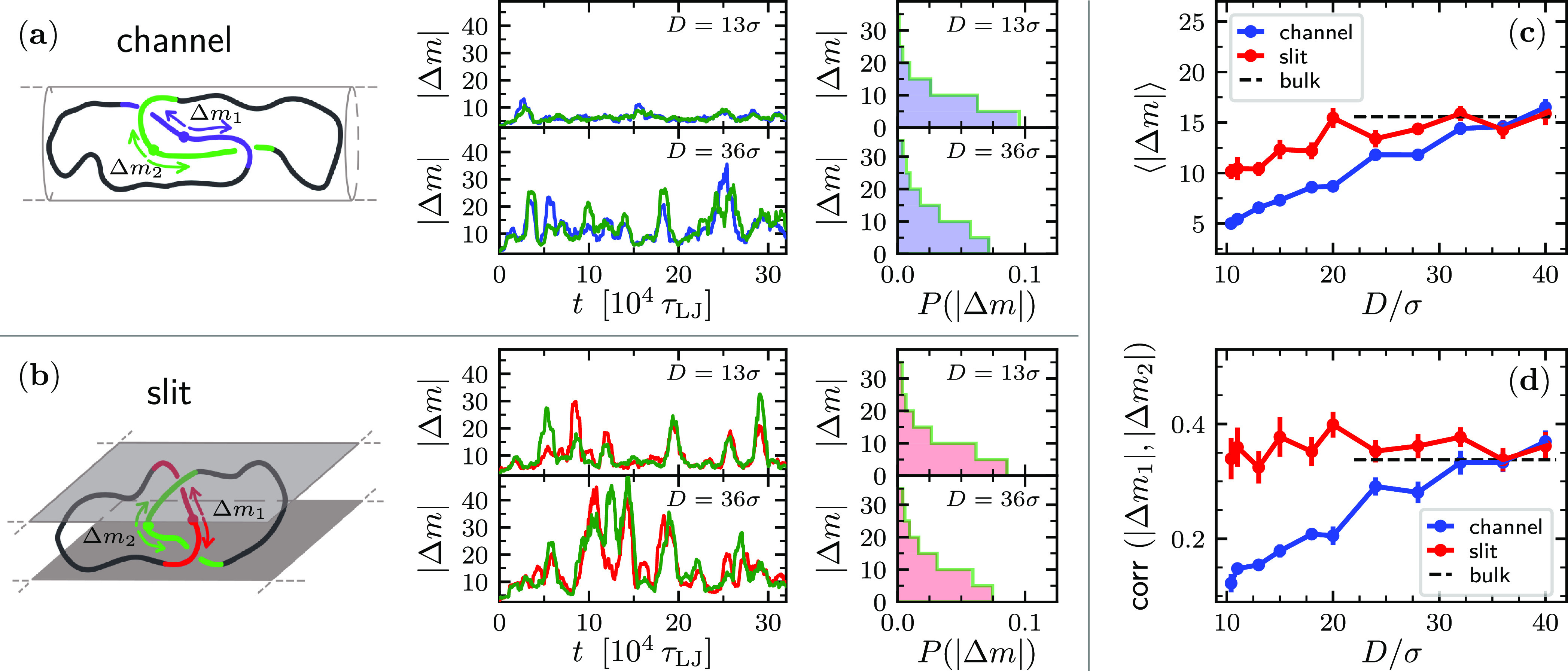
(a,b) The contour migration of the linked region is described via the contour displacements of its two central monomers, *Δm*_1_ (blue, channel; red, slit) and *Δm*_2_ (green) computed for a time lag of 100τ_LJ_. The latter was picked for efficiency, as the associated Δ*m*’s are smaller but still comparable to  in our strongest confinements. The traces show the typical time evolution of |*Δm*| at two different channel (top) and slit (bottom) widths *D*, time-averaged over windows of 10^4^τ_LJ_ for clarity. The absolute value |*Δm*| was considered to discount reversals of migration directionality due, e.g., to ring “flips”, see Figure S3. The side plots show the corresponding probability distributions cumulated over several trajectories. Effect of confinement on the average of |*Δm*| (c) and on the Spearman’s rank correlation coefficient of |*Δm*_1_| and |*Δm*_2_| at equal times (d). The horizontal dashed lines are for the unconstrained (bulk) case. Error bars are the standard deviation of the mean from block analysis.

Second, the contour migration of the two interlocked regions is visibly correlated or coupled in both types of confinement. For a robust measure of the |*Δm*|’s coupling strength, we used a nonparametric association measure, namely the Spearman’s rank correlation coefficient, see Figure S5. The results are given in Figure 2(c,d) and show a stark qualitative difference from one- to two-dimensional confinement. For slits, the degree of correlation of the |*Δm*|’s varies only modestly across the range of explored widths. For channels, instead, the correlation decreases noticeably with increasing confinement. The conclusions are unchanged if a different time lag is used to compute the |*Δm*|’s, see Figure S5(e,f). Overall, the analysis of Figure 2c,d shows that the linked region kinetics differs strongly from channels to slits.

The results above have a common microscopic underpinning. This is illustrated in Figure 3, where the same displacements of Figure 2c,d are plotted as a function of the length of the linked region rather than confinement width. In these plots, the |*Δm*| curves appear to bridge naturally, with deviations comparable to the estimated error bars. The bridging is even clearer for the correlation data, that fall on the same curve regardless of whether the data are for slits or channels. This fact points at  as being a fundamental physical length scale in topologically linked chains and gives to the curves of Figure 3 a character of universality.

**Figure 3:**
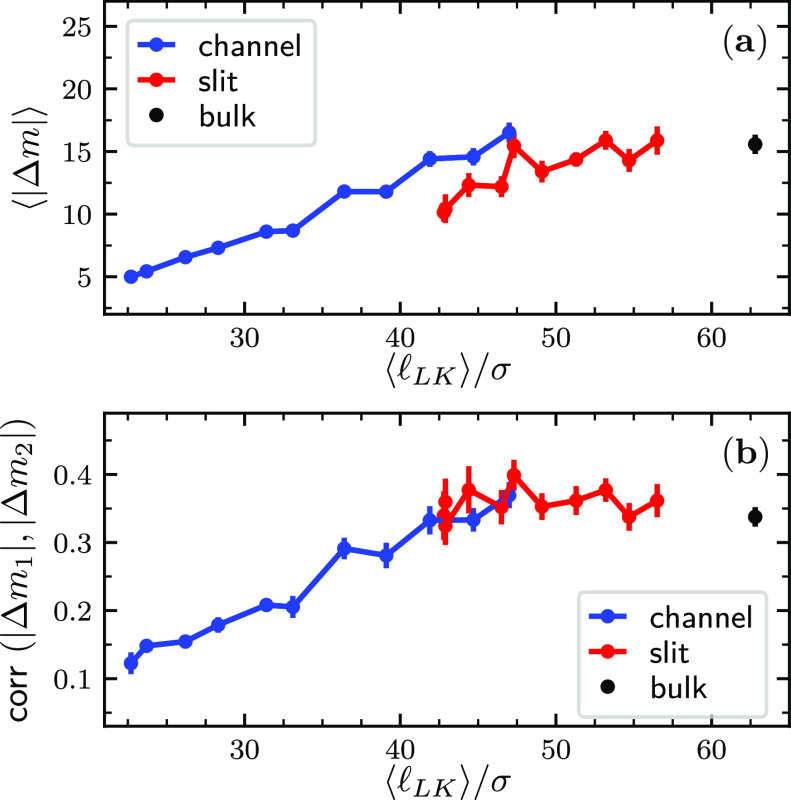
Data of Figure 2c,d are reported as a function of the average contour length of the linked portion. The error bars on the *x*-axis are smaller than the data point symbols.

The |*Δm*|’s amplitude and correlation data of Figure 3 capture the extent to which the two linked portions drag each other due to their topological locking. The observed “topological friction” decreases as the growing confinement makes the linked region shorter and tighter. This is counterintuitive because the hindrance of the contour sliding of other forms of entanglement, such as knots in pulled chains,^35−43^ increases with the tightness of the knots.

The coupling strength thus grows with , and this, we surmise, reflects the additional opportunities for the folds inside the overlapping regions of the two rings to interdigitate and create entangled strand juxtapositions. Not dissimilarly from the obstacles encountered by polymers in a gel,^44^ the juxtapositions affect the rings’ kinetics too. It appears that it is the numerosity of such entanglements, more than their tightness, that builds an effective mechanism for transmitting the contour motion across the two interlocked regions.

This conclusion is supported by a direct investigation of the “rotational motion” coupling of the two rings in tight channels. We adapted the method of ref (45) to compute the correlation of the effective rotational motion of progressively longer arcs centered on the innermost monomers of the two rings (which in tight channels become viable proxies for the central monomers of the linked portion, see Figure S8). The analysis yielded a correlation strength analogous to that obtained for the |*Δm*|’s and further showed that the coupling becomes negligible outside the linked region, see Figure S8.

We conclude the characterization of the kinetics by noting that the diffusive migration of the linked region over the entire rings’ contour is much slower than other global relaxation modes such as the longitudinal self-diffusion and the rings’ reorientation,^46^ see Figures S6 and S7. The latter was characterized using the terminal autocorrelation function (TACF)^47^ and measures the average reorientation time of all diameter vectors joining two monomers at half-ring separation.

A natural question is whether the correlated motion of the rings’ contour is informative about the relaxation kinetics of other polymeric systems where entanglement is not permanently locked into a topologically linked state.

To this end, we cut open one of the two rings in a position diametrically opposite to the linked region central monomer, thus turning the topological link into a physical one without affecting the interlocked region. We then monitored the evolution of the physical link for several trajectories and measured the average unthreading time, τ_unthreading_, which is the time required by the cut ring to disengage from the other intact ring.

The confinement dependence of τ_unthreading_ is shown in Figure 4. One notes that τ_unthreading_ grows rapidly with increasing channel confinement and thus is decoupled from conventional modes of internal relaxation of individual rings.^48−57^

**Figure 4:**
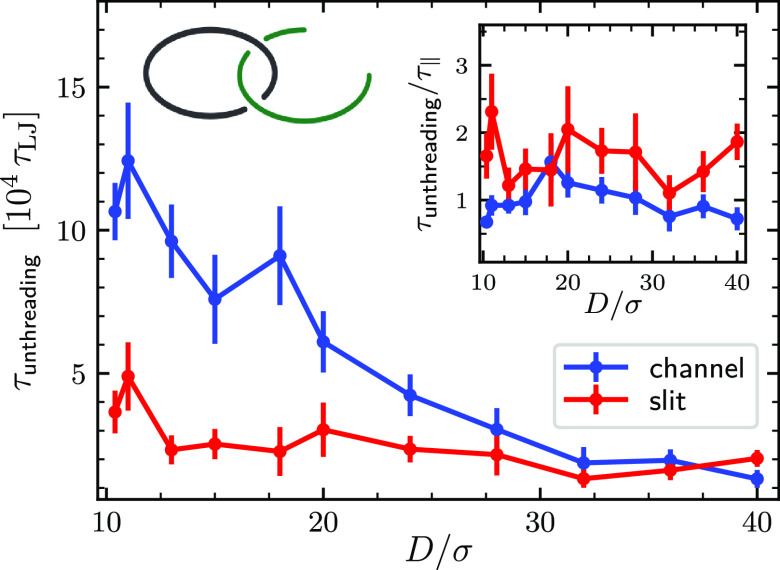
*D*-dependence of τ_unthreading_, the average unlinking time required to observe the separation of the entangled chains after one of the catenated rings is cut open at a site diametrically opposite to the linked region. The inset shows the ratio τ_unthreading_/τ_∥_ versus *D*, where τ_∥_ is the typical time required by two non-interacting rings to diffuse away from each other over a distance equal to the characteristic longitudinal size of the system, see the SI.

A key point is that the unthreading process is about an order of magnitude faster than the aforementioned migration time of the linked region over the entire rings’ contours. For instance, for the strongest considered confinement, the unlinking time was about 3.6 · 10^4^τ_LJ_ for slits and 10.7 · 10^4^τ_LJ_ for channels, while the global migration times are 2.2 · 10^5^τ_LJ_ and 11.6 · 10^5^τ_LJ_, respectively.

We instead found that, for both slit and channel confinement, the unlinking times are comparable to another relevant metric time scale, τ_∥_, which is the time required by two rings to diffuse away from each other over a distance equal to the characteristic longitudinal size of the system, see Figure S6. Indeed, the inset of Figure 4 shows that at all considered slit and channel confinements, τ_unthreading_ remains comparable to τ_∥_.

The results clarify that the relevant relaxation mode governing the unthreading kinetics is the longitudinal diffusive motion of the two chains and not the global migration process of the interlocked regions of the Hopf link, which would be much slower. From this, we conclude that considering interlocked polymers with circular topology is important to isolate the manifestations of topological friction, as the latter can be overriden by competing relaxation mechanisms in the case of linear chains.

In summary, by harnessing a recently developed method to identify reciprocally entangled chain portions,^24,25^ we carried out a first systematic study of how slit and channel confinement affects the static and kinetics of catenated rings. We observed that the contour motion of the two interlocked regions is coupled by an effective drag or topological friction. Differently from other systems^35−43^ the topological friction decreases as the interlocking is made tighter, but also shorter, by confinement. Finally, from the major changes of relaxation kinetics observed after one of the rings is cut open, we conclude that the manifestations of topological friction are best isolated in interlocked polymers with circular backbones (links).

Accordingly, it would be worthwhile to extend future theoretical and experimental considerations to other intermolecular linking topologies beyond the Hopf link which ought to present additional layers of metric and kinetic complexity.

## Supporting Information Available

MODEL and setup of MD simulations, detection of linked portion, metric scaling of confined Hopf-linked and isolated rings, size of linked portion at different confinements, contour migration of linked region, and characteristic relaxation times (PDF)
