# Supplementary material for: Topological Friction and Relaxation Dynamics of Spatially Confined Catenated Polymers
Source: ACS Macro Lett. 2021 Dec 13;11(1):1–6. doi: 10.1021/acsmacrolett.1c00594 (PMC8772382; doi:10.1021/acsmacrolett.1c00594)
Supplement: Supplementary file 1 — mz1c00594_si_001.pdf [file mz1c00594_si_001.pdf]

Supporting Information

"Topological friction and relaxation dynamics of spatially-confined catenated polymers"

Giulia Amici,[1] Michele Caraglio,[2] Enzo Orlandini,[3] and Cristian Micheletti[1]

[1] *Scuola Internazionale Superiore di Studi Avanzati - SISSA, via Bonomea 265, 34136, Trieste, Italy*

[2] *Institut für Theoretische Physik, Universität Innsbruck, Technikerstraße 21A, A-6020 Innsbruck, Austria*

[3] *Department of Physics and Astronomy, University of Padova, Via Marzolo 8, I-35100 Padova, Italy*

MODEL AND SETUP OF MD SIMULATIONS

We modelled each ring as a semiflexible chain of $N = 360$ beads with nominal diameter σ and with standard potential energy terms as in, e.g. refs. [1, 2]. The interaction of the beads with the structureless walls of the confining slit or channel was treated with the same truncated and shifted Lennard-Jones potential used for the steric interaction of the beads. The beads' mass and friction coefficient for the Langevin dynamics were set to standard values as in ref. [3]; the corresponding diffusion coefficient for a single bead was equal to $\mathcal{D}_0 = 2$, expressed in simulation units.

For both slit and channel confinement we considered 11 distinct confinement widths, D, between 10.4σ and 40σ. Furthermore, we also considered an unconstrained (bulk) case.

For each D we carried out 10 Langevin dynamics simulations starting from a nearly fully elongated Hopf link, which we relaxed for a timespan of $10^5\tau_{\rm LJ}$ for slits and $2 \cdot 10^5\tau_{\rm LJ}$ for channels, where $\tau_{\rm LJ}$ is the characteristic Lennard-Jones time. Each simulation then entered the production run with typical duration of $10^7\tau_{\rm LJ}$, which is much larger the slowest relaxation times of the system, see Fig. S6.

Metric and topological observables were averaged over uncorrelated configurations sampled from the production runs. Statistical errors were estimated by first computing the average separately for each independent run and then calculating the error of the mean.

For the analysis of the dynamics of the central monomers of the linked portion we analysed a single excerpt from one of the trajectories at each considered confinement. The duration of the excerpts ranged from $10^6\tau_{\rm LJ}$ to $4 \cdot 10^6\tau_{\rm LJ}$.

These durations, though shorter than a full trajectory for computational requirements, are much larger than the reorientation time at any confinement, see Fig. S7a. They are also comparable to the global contour migration time in the tightest channels and exceed it in all other cases, see Fig. S6b. The ring conformations and the interlocked regions are thus renewed several times during the analysed trajectories.

DETECTION OF THE LINKED PORTION

The linked portion was established with the method introduced in ref. [1]. This employs a top-down search of the shortest pairs of subchains (one for each ring) that yield the same topology of the original pair of rings, in this case the Hopf link topology, once closed with suitable auxiliary arcs. The latter are constructed by prolonging one portion along the outgoing rays connecting its termini to the center of mass of the partner portion, and then bridging each pair of rays "at infinity". The length of the linked portion, $\ell_{\rm LK}$, is the sum of the lengths of the two interlocked regions, one in each ring. The method thus combines topological concepts with geometrical ones, and can be used to locate the interlocked region of mutually entangled chains, being they either open, as in the case of dense solutions or melts of linear chains [1], or closed, as for the considered Hopf-linked rings.

To speed up the combinatorial exploration of the subchains defining the physical link we preconditioned the top-down search as follows. *Channel confinement:* We consider the projections of the two rings along the channel axis and identify their logitudinal overlap. For each ring, we next identify the shortest uninterrupted subchain that covers the overlapping region. These subchains, one per ring, are taken as starting point for the top-down search of the linked portion. *Slit confinement:* We randomly pick 500 in-plane directions and for each of them we identify the subarcs as described above for the channel case. Among all the random directions we only retain those yielding the shortest summed length of the two subchains. These are then taken as the starting point for the top-down search of the linked portion.

METRIC SCALING OF CONFINED HOPF-LINKED AND ISOLATED RINGS

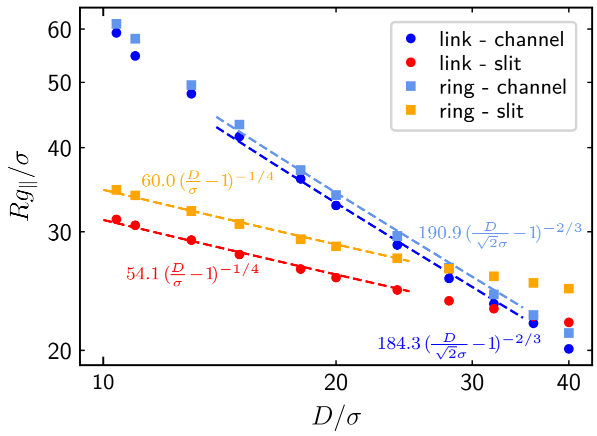

FIG. S1. Longitudinal component of the radius of gyration, $Rg_{\parallel}$ as a function of the scaled confinement width D/σ for Hopf-linked rings of $N = 360$ beads each and for an equivalent single ring of $N = 720$ beads. The plot is in log-log scale and the dashed lines are best fits to the data using the theoretical scaling exponents in the theoretically-predicted range of validity of the anisometric scaling regime [4]. The arguments of the fits are the effective widths of slits and channels. For channels, the definition of the effective width accounts for double-strand occupancy of the confining cylinder: $D_{\rm eff}/\sigma = D/(\sqrt{2}\sigma) - 1$, as suggested in ref. [5]. For slits $D_{\rm eff}/\sigma = D/\sigma - 1$

.

SIZE OF THE LINKED PORTION AT DIFFERENT CONFINEMENTS

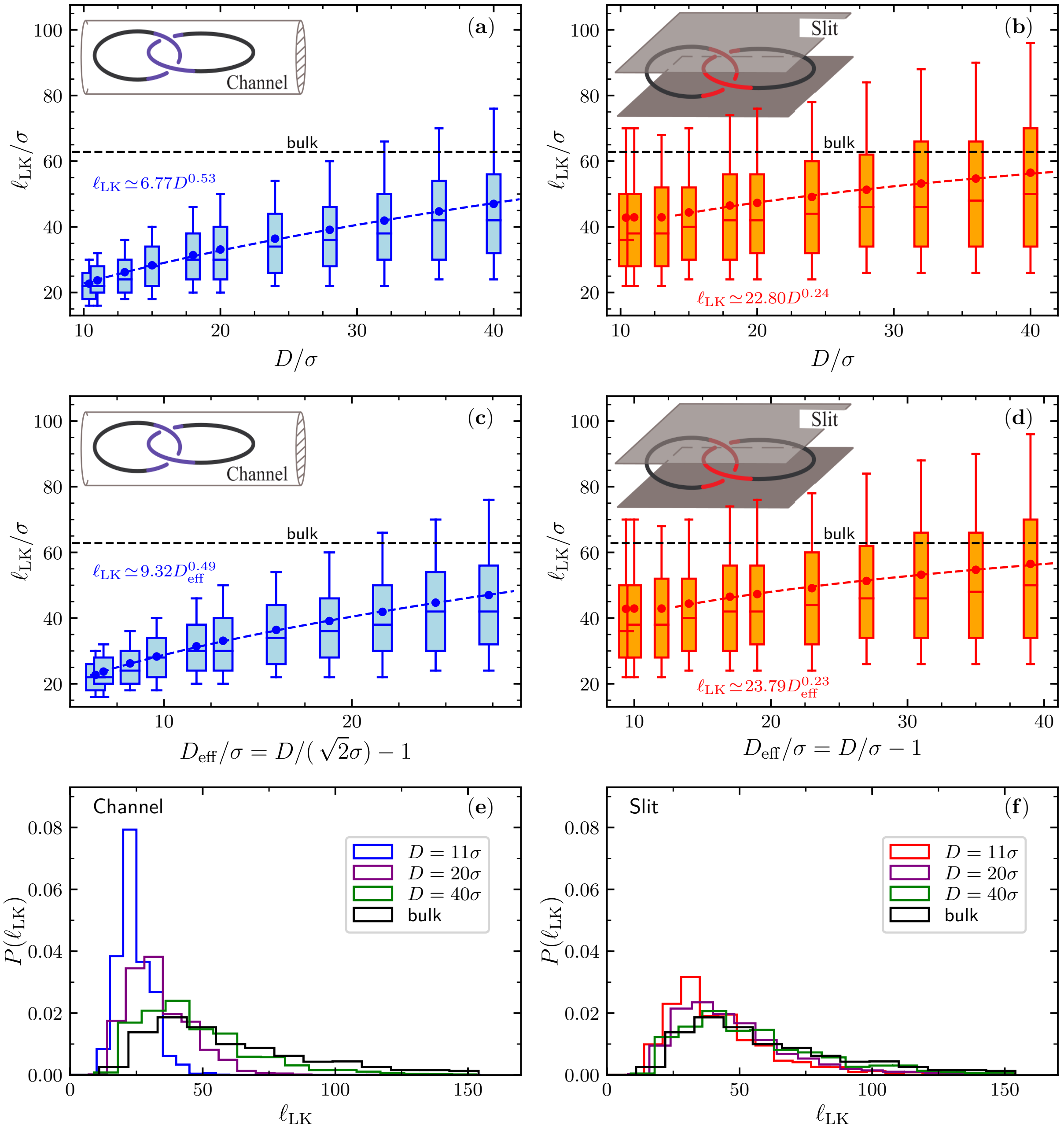

FIG. S2. **(a,b)** Box-whisker plots (point, average; centre line, median ; box limits, upper and lower quartiles; whiskers, 10th and 90th percentiles) of the linked portion length, $\ell_{\rm LK}$, for different channel and slit widths, D. The black dashed lines represent the average value of $\ell_{\rm LK}$ in the bulk or unconstrained case. The blue dashed line in panel (a) is the best fit to the power law $f(x) = ax^b$ over the whole range of values of D for the channel. The red dashed line in panel (b) is a fit to the slit data in the range $D \geq 15\sigma$, that is excluding the narrowest widths at which $\ell_{\rm LK}$ levels off. **(c,d)** Same as in panels (a,b) but with respect to the effective slit and channels widths, $D_{\rm eff}$, see also caption of Fig. S1. **(e,f)** Distributions of $\ell_{\rm LK}$ at different channels and slit confinements, respectively. The $\ell_{\rm LK}$ distribution for the bulk or unconstrained case is also provided for reference.

CONTOUR MIGRATION OF THE LINKED REGION

We studied the stochastic contour migration of the interlocked regions of the two linked rings, which we identified with the algorithm described in the previous section. Typical examples of the migration kinetics are shown in the band kymographs of Fig. S3(a,b). The results are for channels and slits of width $D = 10.4\sigma$. The kymographs portray the time evolution of the stretch of monomers that define the interlocked or linked regions in each of the two rings. Thus, the time-dependent thickness of the curves is related to the length of the linked portion, $\ell_{\rm LK}$, which is the sum of the lengths of the two interlocked regions.

The motion of the two interlocked regions, is tracked via the chain index of their central monomers, $m_k(t)$, where $k = 1, 2$ is the ring index. Specifically, we compute the contour displacements $\Delta m_k(t) = m_k(t + \Delta t) - m_k(t)$ for a timelag $\Delta t = 100\tau_{LJ}$. This value has been chosen since it gives a contour displacement Δm smaller but still comparable with the size of the linked portion for links in the smallest channel. In Fig. S3(c,d) we show the distribution of the Δm's for two different levels of confinement $D = 10.4\sigma$ and 36σ and, for reference, for the unconstrained (bulk) case, too. Gaussian fits are superimposed to the histograms.

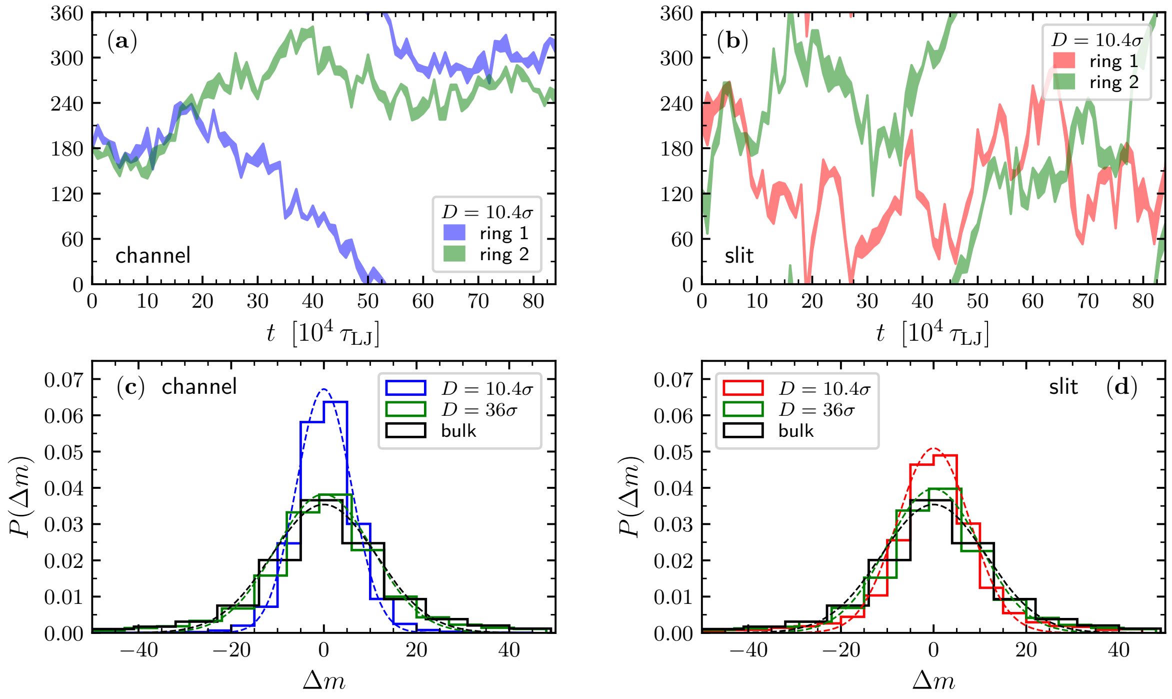

FIG. S3. **(a,b)** Kymographs showing a typical time evolution of the chain index of the monomers belonging to the linked portion of ring 1 and ring 2 confined in channels (a) and slits (b) of width $D = 10.4\sigma$. Notice the occasional reversals of directionality of the motion in the two rings (e.g. for $20 < t < 40$ $[10^4\tau_{\rm LJ}]$ for panel (a)), which are consistent with e.g. ring flips in correspondence of the linked region. **(c,d)** Probability distributions of the Δm's, computed with time lag $\Delta t = 100\tau_{LJ}$, and their Gaussian fits (dashed curves) for two different confinement levels ($D = 10.4\sigma$ and $D = 36\sigma$) for channels (c) and slits (d). The corresponding distribution and fit for Hopf links in the bulk is reported in both panels as reference.

To better investigate the contour migration process, we integrated the contour displacements of the central monomers of the linked portions over time intervals of increasing duration.

The mean square displacements Δm show an approximate linear dependence over time intervals Δt, see Fig. S4(a,b). This supports the diffusive character of the contour migration process. The periodic nature of the ring contour can be taken into account explicitly, by"folding back" the Δm's in the $[-N/2, N/2]$ interval. With such proviso, the mean square displacements is described by a diffusion along the periodic ring contour, see Fig. S4(c,d):

$$\langle \Delta m^2 \rangle = \frac{1}{\sqrt{4\pi \mathcal{D}_{\rm LK} \Delta t}} \sum_{p=-\infty}^{\infty} \int_{-N/2}^{N/2} {\rm d}x\, x^2\, e^{-\dfrac{(x + p\,N)^2}{4\mathcal{D}_{\rm LK}\Delta t}} \,. \tag{S1}$$

The above expression was used to fit the (folded) Δm vs Δt data obtaining the effective diffusion coefficient for the

contour motion of the linked regions, $\mathcal{D}_{\rm LK}$, at all considered confinements, see Fig. S4(e,f).

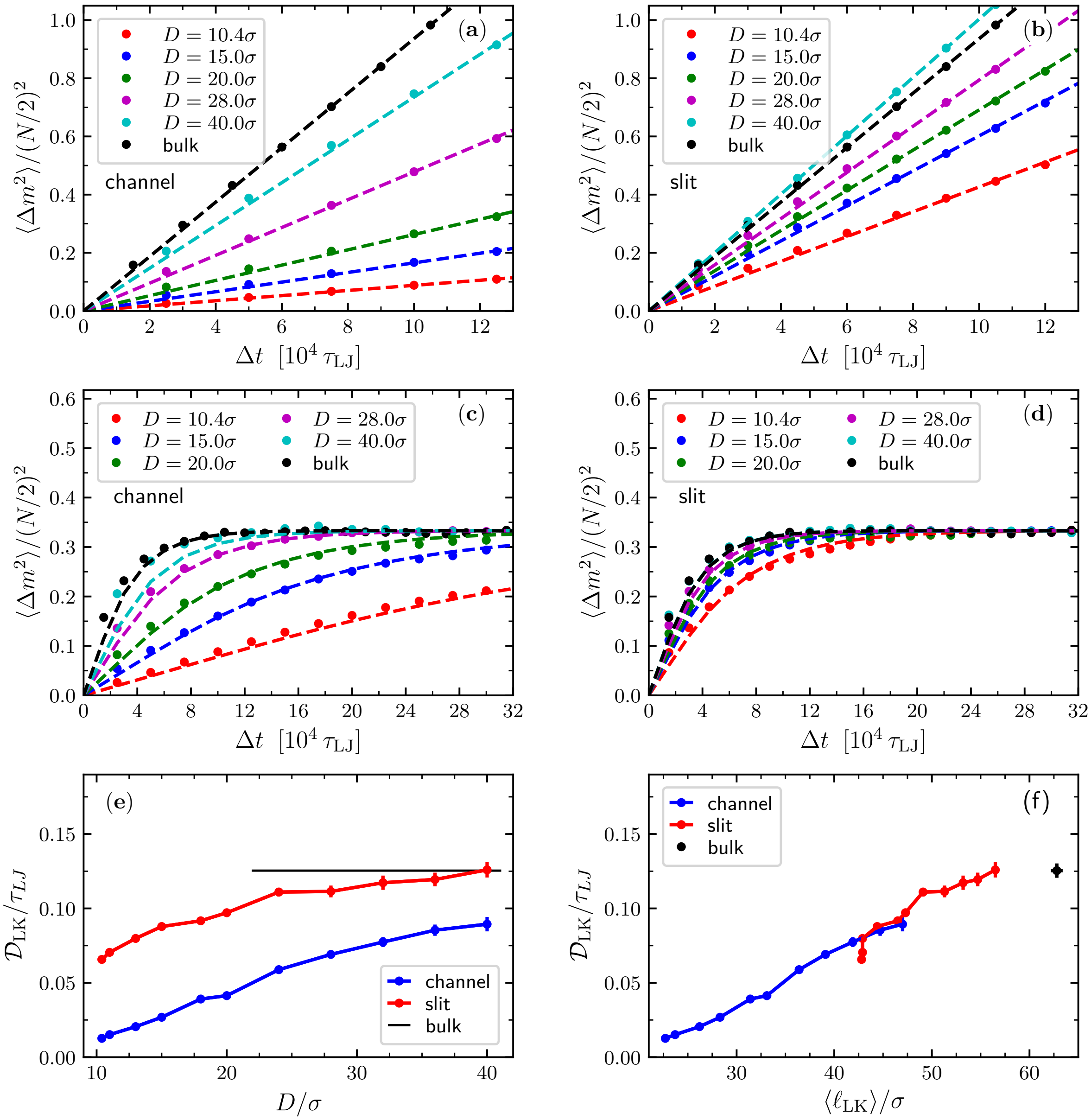

FIG. S4. **(a,b)** Mean square contour displacement $\langle \Delta m^2 \rangle$ of the central monomer of one of the two linked portions. The data are averages over both linked regions and various trajectories for each indicated channel and slit widths, D. **(c,d)** Same as (a,b) but the mean square displacement and the fitting curves now take into account the periodicity of the ring contour, see Eq. S1. **(e,f)** Diffusion coefficient, $\mathcal{D}_{\rm LK}$ of the linked portion as a function of (e) the confinement width D and of (f) the linked portion $\langle \ell_{LK} \rangle$ for slits and channels. The diffusion coefficient $\mathcal{D}_{\rm LK}$ was obtained from the fits of panels (b,c).

The average values of $|\Delta m|$ taken at two different time lags, $\Delta t = 100\tau_{LJ}$ and $\Delta t = 200\tau_{LJ}$ and reported as a function of the average size of the linked portion, are compared in Fig. S5(a,b) for both channels and the slits.

To quantify the observed coupling between the two intertwined portions (one for each ring) we analysed the correlation of $|\Delta m_1|$ and $|\Delta m_2|$ over a given trajectory. A scatter plot of the $|\Delta m|$'s for the two rings is reported in Fig. S5(c) with the corresponding density plot in overlay. Because the data points are inhomogeneously distributed in one quadrant of the Cartesian plane, a linear regression analysis is not suitable to assess their correlation, for the lack of null model. We thus resorted to the non-parametric Spearman's correlation analysis on the pair $(|\Delta m_1|, |\Delta m_2|)$, which harnesses the fact that, regardless of the distribution of origin, the one of the ranks is uniform. The Spearman correlation coefficient is defined as the Pearson correlation coefficient between the rank indices of the datapoints in

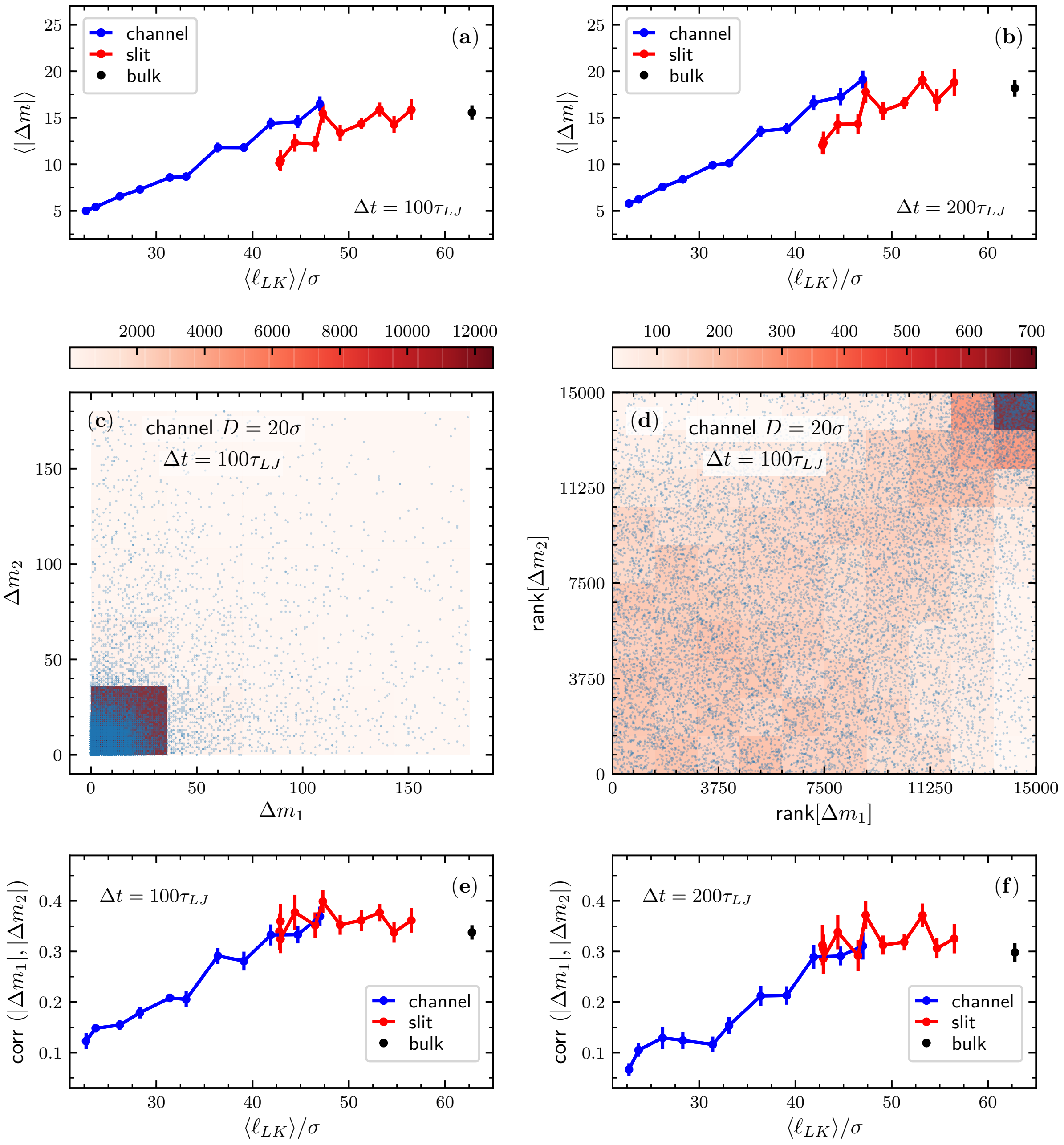

FIG. S5. **(a,b)** Average values of Δm for the indicated different time lags, Δt, plotted as a function of the size of the linked portion for both slit- and channel-like confinement. The black datapoint represents the reference data of the bulk case. Averages are obtained by considering both the chains of the concatenated rings. **(c)** Scatter plot of $|\Delta m_1|$ vs $|\Delta m_2|$, with overlaid density heatmap. **(d)** Scatter plot of the ranks of $|\Delta m_1|$ vs. the ranks of $|\Delta m_2|$, with overlaid density heatmap. **(e,f)** Spearman's rank correlation coefficient of $|\Delta m_1|$ and $|\Delta m_2|$ at different confinements are plotted as a function of $\langle \ell_{\rm LK} \rangle$. Data for panel (e) are for $\Delta t = 100\tau_{LJ}$, and those of panel (f) are for $\Delta t = 200\tau_{LJ}$.

the two sets, namely:

$$\mathrm{corr}(|\Delta m_1|, |\Delta m_2|) = \frac{\mathrm{cov}\left[\mathrm{rank}(|\Delta m_1|), \mathrm{rank}(|\Delta m_2|)\right]}{\sigma_{\mathrm{rank}(|\Delta m_1|)}\, \sigma_{\mathrm{rank}(|\Delta m_2|)}}\,, \tag{S2}$$

where $\mathrm{cov}\left[\mathrm{rank}(|\Delta m_1|), \mathrm{rank}(|\Delta m_2|)\right]$ and $\sigma_{\mathrm{rank}(|\Delta m_1|)}$, $\sigma_{\mathrm{rank}(|\Delta m_2|)}$ are the covariance and the standard deviations of the rank variables, respectively. The scatter plot of the ranked variables are shown in Fig. S5(d), with the corresponding density plot in overlay. The corresponding Spearman's rank correlation coefficient is about 0.35.

The values of the Spearman's rank correlation coefficients are summarised in Fig. S5(e,f) for different confinements and for two different choices of the time lag Δt.

CHARACTERISTIC RELAXATION TIMES

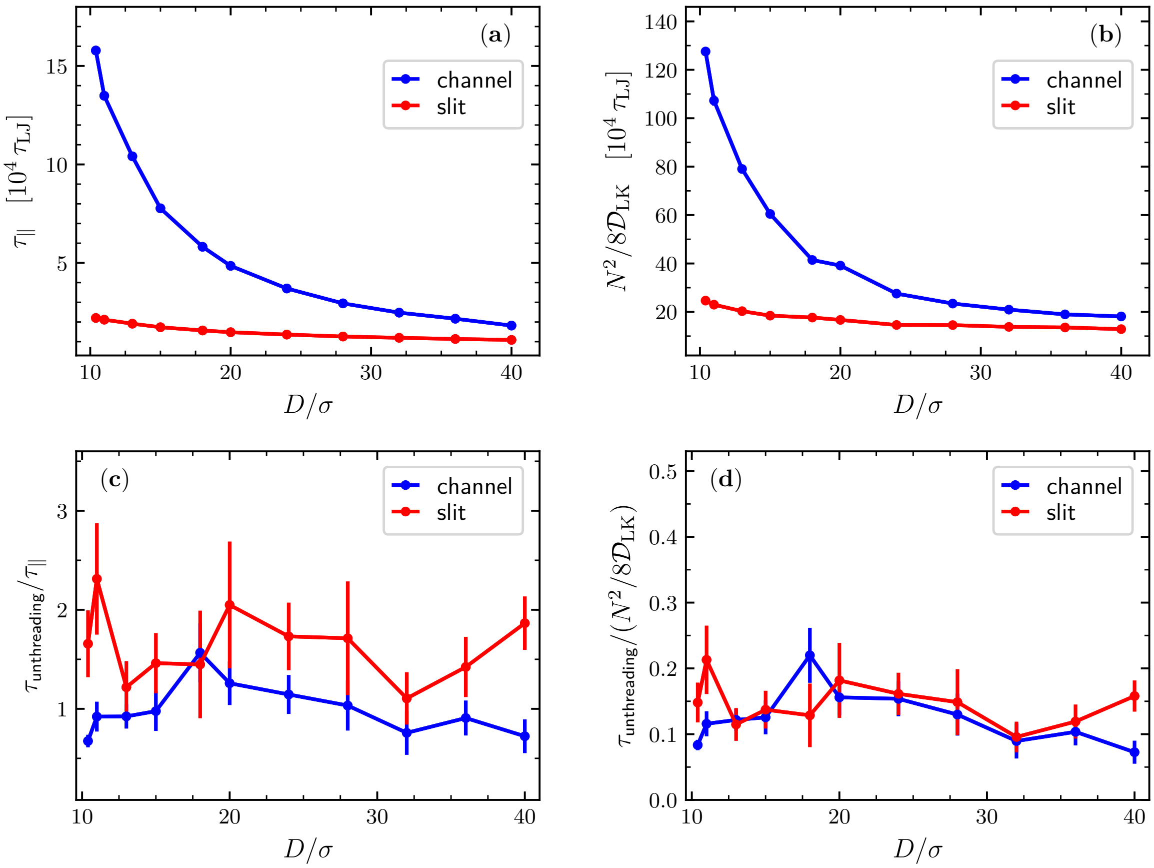

FIG. S6. **(a)** Confinement dependence of $\tau_\parallel$, which is the time required by two non-interacting rings of $N = 360$ beads to diffuse away from each other over a longitudinal distance equal to $Rg_\parallel$. The curves were obtained from the $Rg_\parallel$ data of Fig. S1, from a ring's diffusion coefficient $\mathcal{D} = \mathcal{D}_0/N$ and by taking into account the one- and two-dimensional nature of the longitudinal stochastic motion in channels and slits, respectively. $\mathcal{D}_0$ is the diffusion coefficient of a single monomer. **(b)** Confinement dependence of the time required by the central monomer of the linked region to diffuse to a diametrically opposite point on the ring contour, that is a point that is $N/2$ monomers away. Here $\mathcal{D}_{LK}$ is the diffusion coefficient of the linked portion as obtained in Fig. S4. **(c,d)** Ratios of the unthreading time (see main text) with the relaxation times reported in panels (a) and (b) respectively.

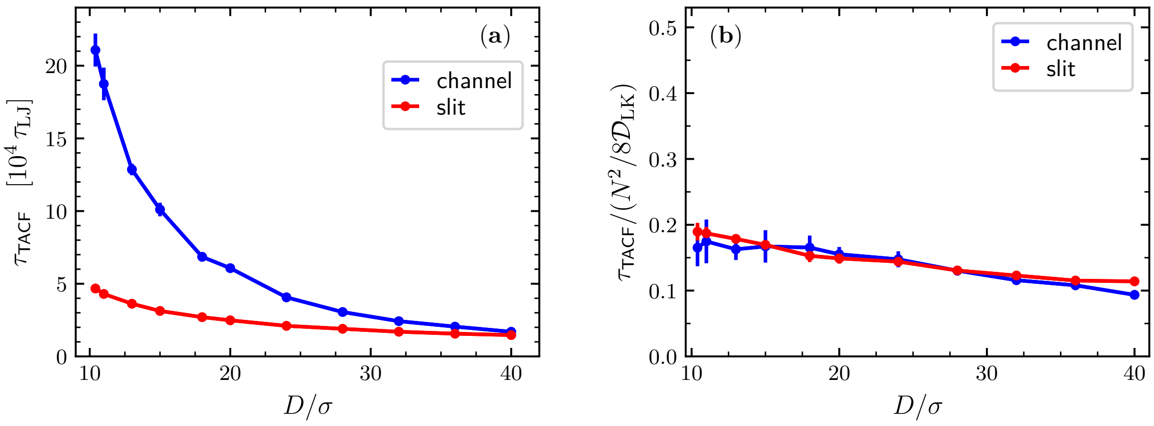

FIG. S7. **(a)** Confinement dependence of the rotational autocorrelation time [6, 7], which was computed as the characteristic timescale of the terminal autocorrelation function, τ_{TACF}. Following Ref. [7], the latter was measured by considering the reorientation time of all the diameter vectors, that is the distance vectors of two monomers at half-ring separation. **(b)** Ratio of τ_{TACF} with the global contour migration time, which is the time required by the central monomer of a linked portion to move to a diametrically opposite monomer on the same ring, see Fig. S6.

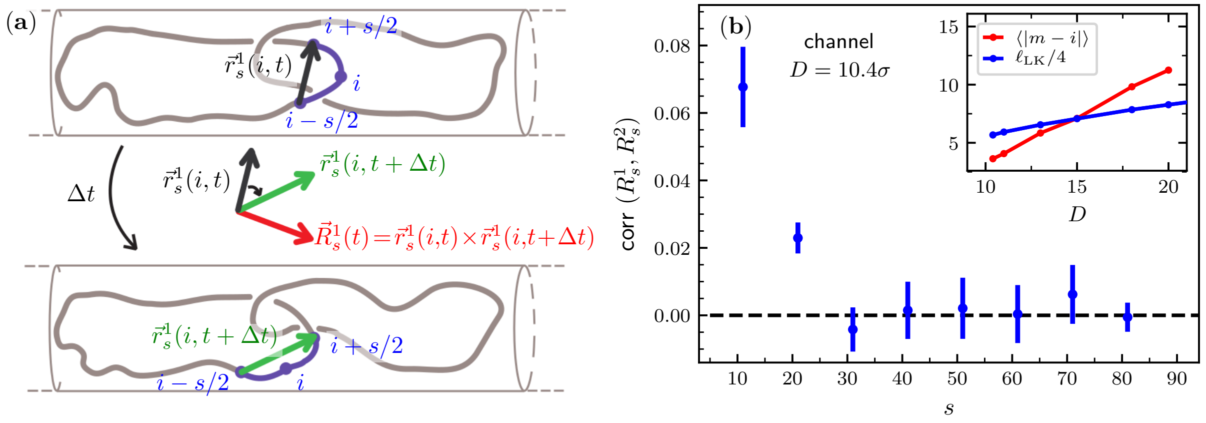

FIG. S8. **(a)** Alternative characterization of the kinetic coupling of the interlocked regions. Inspired by Halverson *et al.* [8] we considered the "rotational" correlation of the end-to-end vector of segments centered at the innermost monomer, or apexes, of the two rings. The magnitude of the rotation for ring $k = 1, 2$ is computed as $R^k_s(t) = |\mathbf{r}^k_s(i,t) \times \mathbf{r}^k_s(i, t+\Delta t)|$, where $\mathbf{r}^k_s(i,t)$ is the end-to-end vector of a segment of length s centered on the innermost monomer, i, of the ring, see sketch in panel (a). In tight channels the innermost monomer, i is a viable proxy for the central monomer of the linked portion, m. This is illustrated in the inset of panel (b) where the contour distance of the two monomers $|m - i|$ is seen to fall below the semilength of the linked portion in each ring, $\ell_{\text{LK}}/4$, at sufficiently small channel widths, D. **(b)** Pearson correlation between R^1_s and R^2_s at $\Delta t = 100\tau_{LJ}$ in narrow channels of width $D = 10.4\sigma$.

[1] M. Caraglio, C. Micheletti, and E. Orlandini, "Physical links: defining and detecting inter-chain entanglement," Scient. Rep. **7**, art no. 1156 (2017).
[2] G. Amici, M. Caraglio, E. Orlandini, and C. Micheletti, "Topologically linked chains in confinement," ACS Macro Lett. **8**, 442 (2019).
[3] K. Kremer and G. Grest, "Dynamics of entangled linear polymer melts: A molecular dynamics simulation," J. Chem. Phys. **92**, 5057 (1990).
[4] Y. Wang, D. R. Tree, and K. D. Dorfman, "Simulation of DNA extension in nanochannels," Macromol. **44**, 6594 (2011).
[5] Y. Jung, C. Jeon, J. Kim, H. Jeong, S. Jun, and B.-Y. Ha, "Ring polymers as model bacterial chromosomes: confinement, chain topology, single chain statistics, and how they interact," Soft Matt. **8**, 2095 (2012).
[6] M. Müller, J. Wittmer, and M. Cates, "Topological effects in ring polymers: A computer simulation study," Physical Review E **53**, 5063 (1996).
[7] A. F. Katsarou, A. J. Tsamopoulos, D. G. Tsalikis, and V. G. Mavrantzas, "Dynamic heterogeneity in ring-linear polymer blends," Polymers **12**, 752 (2020).
[8] J. D. Halverson, W. B. Lee, G. S. Grest, A. Y. Grosberg, and K. Kremer, "Molecular dynamics simulation study of nonconcatenated ring polymers in a melt. ii. dynamics," The Journal of chemical physics **134**, 204905 (2011).
